# Three-dimensional studies of pathogenic peptides from the c-terminal of *Trypanosoma cruzi *ribosomal P proteins and their interaction with a monoclonal antibody structural model

**DOI:** 10.1186/1757-5036-2-4

**Published:** 2009-05-27

**Authors:** Osvaldo A Martín, Myriam E Villegas, Carlos F Aguilar

**Affiliations:** 1 Instituto de Matemática Aplicada de San Luis, CONICET, Ejército de los Andes 950, primer piso, 5700, San Luis, Argentina; 2 Departamento de Física de la Universidad Nacional de San Luis, Ejército de los Andes 950, Bloque II, segundo piso, 5700, San Luis, Argentina; 3 Laboratorio de Biología Molecular Estructural, Facultad de Química, Bioquímica y Farmacia, Universidad Nacional de San Luis, Ejercito de los Andes 950, Bloque I, 5700 San Luis, Argentina

## Abstract

The acidic C-terminal peptides from *Trypanosoma cruzi *ribosomal P proteins are the major target of the antibody response in patients suffering Chagas chronic heart disease. It has been proposed that the disease is triggered by the cross-reaction of these antibodies with the second extra cellular loop of the β1-adrenoreceptor, brought about by the molecular mimicry between the acidic C-terminal peptides and the receptor's loop. To improve the understanding of the structural basis of the autoimmune response against heart receptors, the 3-dimensional structure of the C-terminal peptides of *Trypanosoma cruzi *ribosomal proteins P0 (EDDDDDFGMGALF) and P2β (EEEDDDMGFGLFD) were solved using the Electrostaticaly Driven MonteCarlo method. Their structures were compared with the second extra-cellular loop of our homology model of human rhodopsin and the existing experimental NMR structures of the C-terminal peptides from human P0 (EESDDDMGFGLFD) and from *Leishmania braziliensis *P0 (EEADDDMGFGLFD). Docking of *Trypanosoma cruzi *peptides P0, P2β and human rhodopsin loop into our anti-P2β monoclonal antibody homology model allowed to explore their interactions.

The solution structure of peptides P0 and P2β can be briefly described as a bend. Although the global conformations of the peptides are not identical they shared a common region of four residues (3 to 6) that have a similar structure. The structural alignment of the five peptides also showed a surprising conformational similarity for the same residues. The antibody model and docking studies revealed a most remarkable feature in the active site, a positively charged, narrow and deep cavity where the acidic residues 3 to 6 were accommodated. These results suggest that the most important elements in the molecular peptide recognition by the antibody may be the shape of the loop and the presence of negative charges in positions 3–5 (P0, P2β) or a negative charge in position 4 (rhodopsin loop). This work describes clearly the interactions of the structural elements involved in the autoimmune mechanism of anti-P auto-antibodies cross-reaction and stimulation of the β1-adrenoreceptor and the visual pigment rhodopsin. Results from this study could lead eventually to the development of treatments to abolish receptor mediated symptoms in Chagas. PACS code: 87.15.-v

## 1. Introduction

The Chagas disease or American tripanosomiasis is a parasitosis produced by the flagellated protozoa *Trypanosoma cruzi*. It is transmitted by the insect vector *Triatoma infestans*. According to a recent report of the Scientific Working Group on Chagas Disease the overall prevalence of human *Trypanosoma cruzi *infection is estimated at 16–18 million cases. Approximately 120 million people or 25% of Latin American inhabitants are at risk of contracting the infection.

People infected with *Trypanosoma cruzi *may suffer cardiac, gastrointestinal or neurological damage. Although disease manifestations vary widely from one area to another, it is estimated that 25–30% of the infected people will progress to irreversible cardiac, oesophageal and colonic pathologies, causing considerable morbidity and mortality [1].

Chagas's chronic heart disease (cChHD) is a myocarditis characterized by arrhythmia, tachycardia and heart failure. In the chronic phase of the disease, patients present antibodies against the acidic rich C-terminal of *Trypanosoma cruzi *ribosomal P proteins [2]. It has been proposed that cChHD is an autoimmune disease produced by the existence of molecular mimicry between the C-terminal of ribosomal P proteins and the second extra cellular loop of the β1-adrenergic receptor (**H26R**) [3].

Ribosomal P proteins are a protein complex which forms the long and protruding region called the *stalk *in the large ribosome subunit. In eukaryotes the P family encompasses protein P0 (34 kD), P1 and P2 (~10 kD) [4]. A third protein, P3, has been found in plants [5]. The number of proteins in the P1 and P2 families varies among species. In mammals, insects and fungus, the complex is formed by P0 and two copies of P1 and P2 [6,7]. Yeast and protozoa like *Trypanosoma cruzi *have five members: P0, P1α, P1β, P2α and P2β [8-10]. All of them have a conserved acidic motif in the C-terminal end and show high sequence identity with the last one hundred amino acids of P0 C-terminal.

**P013 **(EDDDDDFGMGALF) and **R13 **(EEEDDDMGFGLFD) are the peptides corresponding to the last 13 amino acids of *Trypanosoma cruzi *P0 and P2β respectively and a major target of the humoral immune response against the parasite [2].

The active immunization with P0 and P2β recombinant proteins from *Trypanosoma cruzi *has been shown to induce stimulation of the β1-adrenoreceptor in murine models. This activity has been correlated with ventricular arrhythmia, abnormal repolarizations and conduction defects [11]. Moreover, it has been proved that monoclonal antibodies against H26R induce apoptotic effects in cardiomyocites [12]. Another study has associated the dysfunction of the retina, suffered by chagasic patients, with a similar autoimmune mechanism in which anti-P auto-antibodies would cross-react with the visual pigment rhodopsin [13].

A preliminary study of the cross reaction between the anti-P2β monoclonal antibody (mAb17.2) and the β1-adrenergic receptor was done by Smulski et al. [14]. They prove that an antibody induced by a *Trypanosoma cruzi *intracellular protein has the ability to bind a peptide which is part of the extracellular domain of a human cardiac receptor. Although such results include a paragraph about molecular modelling studies describing the manual docking of minimized structures of charged hepta peptides from P2β C-terminal (EDDDMGF) and the second extra cellular loop of the β1 adrenergic receptor (ESDEARRCYN) into an homology model of the antibody, they did not report detailed interaction studies.

The aim of this work is to contribute to a deeper understanding of the molecular basis of Chagas's chronic heart disease through the structural study of the C-terminal peptides and their interaction with the homology model of the anti-P2β monoclonal antibody(mAb17). The solution conformation structures of peptides **P013 **and **R13 **are the result of the global optimization of the all atom force-field ECEPP/3 plus implicit hydration models using the EDMC method (Electrostatically Driven Monte Carlo) [15,16].

The EDMC structures were compared with the experimental NMR structures of human P0 **H13**, PDB code 1S4J (EESDDDMGFGLFD), *Leishmania braziliensis *P0 **A13**, PDB code 1S4H (EEADDDMGFGLFD) [17], and **SEL **(RYIPEGLQCSCGIDYYTLKPEVNNES), the second extra-cellular loop of our human rhodopsin homology model.

The conformational study of the antigen-mAb17.2.2 interaction can be divided in three steps:

1) Conformational search to define the three dimensional structure of **R13 **and **P013**.

2) Homology modeling of rhodopsin and monoclonal antibody **mAb17.2**

3) Molecular docking. The antigen-antibody interaction was analyzed through the docking of the different peptides onto the target antibody **mAb17.2**

## 2. Methods

### 2.1. Conformational search

Electrostaticaly driven Monte Carlo **(EDMC)**. The first step consisted in generating feasible target peptide conformations using the EDMC method. The method combines stochastic Monte Carlo algorithms and the optimization of the electrostatic interactions, whereby the molecule evolves toward conformations in which the charge distribution becomes energetically more favourable.

### 2.2. Evaluation of the Conformational Energy

An all-atom chain representation was used with the ECEPP3 force field [18-22]. Two alternative forms of the potential energy function were used to evaluate the total energy during the conformational search. One, in vacuum corresponding to the ECEPP/3 force field and the other adding a simple solvent interaction model of surface accessibility [23-25].

### 2.3. The Starting Point and conformational search

The amino and carboxyl termini of the target fragment of the P protein molecule were blocked by amino-COCH3 and carboxyl-NH2 groups, respectively. An ensemble of conformations was generated for each peptide starting from **(I) **canonical α-helix, assigning the values (-60°,-40°) to dihedral angles (Φ,Ψ) of each amino acid residue, while keeping all values of ω to 180°. **(II) **polyproline conformation, assigning the values (-80°,150°) to dihedral angles (Φ,Ψ) of each amino acid residue, while keeping all values of ω to 180° **(III) **canonical β-strand, assigning the values (-139°,135°) to dihedral angles (Φ,Ψ) of each amino acid residue, while keeping all values of ω to 180° **(IV) **Random conformation, the simulations were started from two different initial random conformations, for each of these random conformations, *all *backbone and side-chain dihedral angles were chosen randomly between 180.0° and -180.0°, with the exception of the dihedral angles ω of the peptide group, which were always chosen in the *trans *(180.0°) conformation. All backbone and side-chain dihedral angles were allowed to vary freely during the simulations.

### 2.4. pH conformational coupling

Many research groups [22,26-31] have devoted a large amount of effort to develop fast and reliable algorithms provide a solution to the problem of ionization equilibrium. However, the incorporation of these algorithms into conformational searches of short peptides is computationally too time consuming. Here, we used an alternative approach in which the energy of the whole ensemble of conformations, generated in a conformational search, is re-evaluated by considering the problem of ionization equilibrium at a given pH explicitly [32]. This methodology permitted us to compute the average degree of charge to each of the ionizable groups in the sequence. Allowing us to find out the importance and influence of the ionizable groups in the peptides, the influence of solvent polarization effects and electrostatic interactions in the discrimination of the lowest-energy conformation.

The evaluation of the conformational energy follows the procedure previously published [22]. The total free energy E(r_p_, pH) associated with the conformation, r_p_, of the molecule in aqueous solution at a given pH, is defined by considering a three-step thermodynamic process: cavity creation, polarization of the solvent, and alteration of the state of proton binding. This free energy involved in transferring the neutral peptide from the gas phase to the aqueous solution is given by:



Where E_int_(r_p_) is the internal conformational energy of the molecule in the absence of solvent, corresponding to the ECEPP/3 energy of the neutral molecule [18-22]; F_vib_(r_p_) is the conformational entropy contribution calculated by the harmonic vibrational contribution with zero charge [33,34]. F_cav_(r_p_) is the free energy associated with the process of cavity creation when transferring the molecule from the gas phase into the aqueous solution; this term is proportional to the solvent accessible surface of the molecuele. F_solv_(r_p_) is the free energy associated with the polarization of the aqueous solution calculated by using the Multigrid Boundary Element method developed by Vorobjev and Scheraga [31]. F_inz_(r_p_, pH) is the free energy associated with the change in the state of ionization of the ionisable groups due to the transfer of the molecule from the gas phase to the solvent at a fixed pH value. This energy is calculated by using the general multi-site titration formalism [26,28]. This approximation computes the 2^N ^ionization states, where N is the total number of ionisable groups. The present method provides an accurate calculation of the potential of mean forces between ionized groups of the protein and the pK shifts of the ionisable groups as a function of the peptide environment.

### 2.5. Homology Modeling

First, the structure of the **mAb17.2 Fv **domain was modelled with MODELLER [35], using as template the PDB code 1NBV (82% sequence identity). PDB code 1S5I was used to model the heavy chain CDR3 (TYWGQGTLVTVS, 100% sequence identity). BLAST [36] and T-Coffee [37] were used to search for the templates and perform the alignment, respectively.

Second, the model of human rhodopsin was generated in a similar way using the bovine rhodopsin structure as template (PDB 1U19, 93% sequence identity). The quality of the models was assessed by the WHAT_CHECK standalone program [38].

### 2.6. Docking

The docking of peptides **R13**, **P013 **and **SEL **into **mAb17.2 **was done using BIGGER (Biomolecular complex Generation with Global Evaluation and Ranking) implemented on Chemera 2.0 [39]. BIGGER has a soft docking algorithm, an intermediate approach between flexible and rigid docking. The flexibility of the side chains was treated in a crude implicit way by allowing partial superposition of the side chains. Default options were used except for the angular step that was set at 12°. For **R13**, 5000 putative complexes were generated and then clustered using a 2.5 Å RMSD. **P013 **was treated in the same way. About 8000 complexes were generated with full length rhodopsin and then clustered using a 2.5 Å RMSD. An additional score based on the distance from the amino acids of the probe to the amino acids of the CDRs was generated. In order to discard unreal solutions, the best results were analysed visually. Three results were chosen for **rhodopsin**. For the **R13 **and **P013 **peptides the final sets were around 15 possible solutions. The final complexes were minimized using the Polak-Ribiere conjugate gradient method using the AMBER99 force field [40]. The value of the potential energy, visual analysis and the comparison among all the different docking runs allowed the selection of just one ligand-antibody complex. They were submitted, for further refinement, to a simulated annealing protocol using the molecular dynamic software NAMD [41].

### 2.7. Docking Refinement

The complexes obtained from molecular docking were submitted to a regular molecular dynamics simulation with NAMD [41] of 7.5 ns, at 300 K, allowing the whole system to move. This simulation did not show significant changes with respect to unrefined docking results.

The next step was to refine the complexes obtained from the molecular docking, using simulated annealing through molecular dynamics simulation with the software NAMD [41]. The parameters of the simulation were optimized, through shorts runs, allowing all system parameters to move.

The optimized protocol was as follows: the system was solvated using a sphere of TIP3 [42] water model. Spherical boundary conditions were applied to avoid the evaporation of water molecules. The motion of the antibody was restricted, allowing only the CDRs residues to move. A langevin piston [43,44] was implemented to keep the pressure at 1 atm. The regulation of the temperature was enforced by a langevin thermostat [45]. An integration time step of 2 fs was used. The simulation started at 300 K and the whole system was heated up to 600 K by 50 K jumps every 10 ps. Then, the temperature was held at 600 K during 120 ps. Afterwards, the temperature was decreased by 50 K jumps. The first jump was done after 10 ps, the second one after 20 ps, then 40 ps, 60 ps and 100 ps. Then, the system was kept at 300 K during 160 ps. The whole process was iterated three times.

## 3. Results

### 3.1. Peptide structure

**Figure 1:**
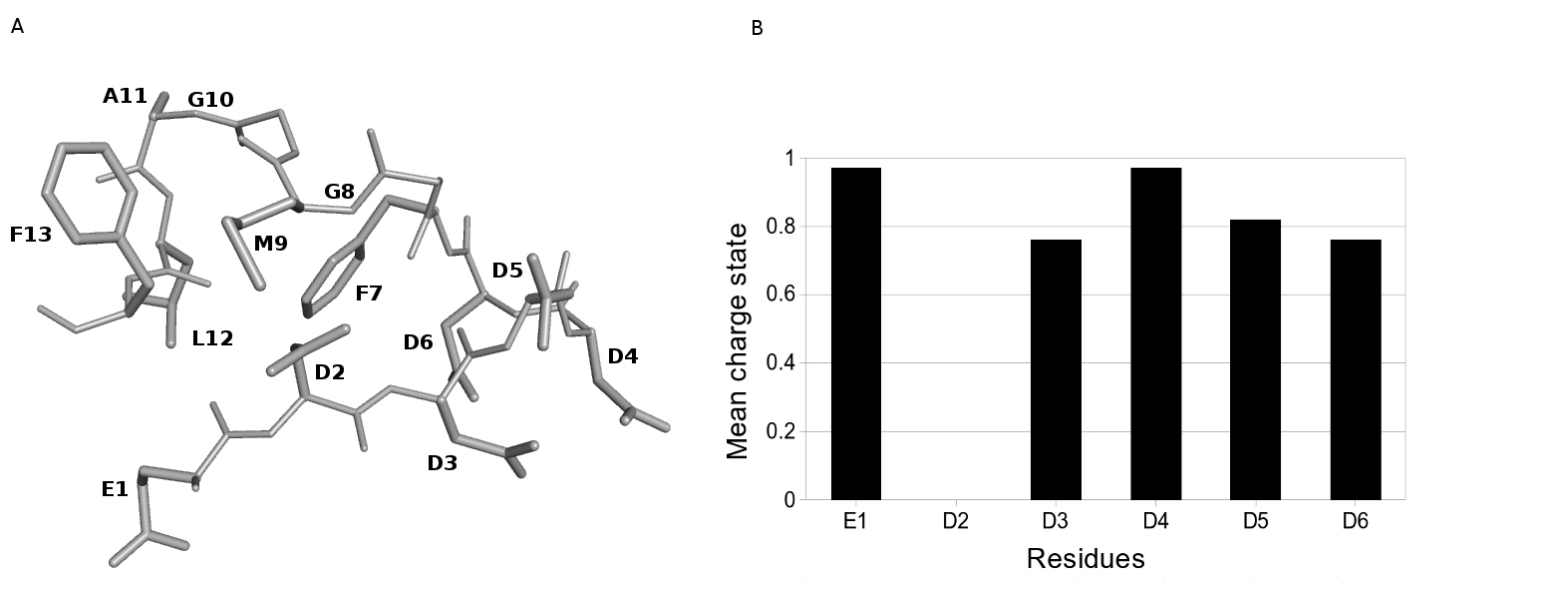
**Structure of P013 peptide**. A) Representation of theoretical minimum energy conformation of P013 peptide. Amino acids are labeled using one-letter code. B) Boltzmann average degree of charge in accord with the conformational pH model. Only D2 is uncharged at pH7.

The structure of **P013 **(EDDDDDFGMGALF) was calculated as explained in the methods section. The whole conformational search carried out produced almost 12000 conformations. The lowest energy conformation, shown in Figure 1a, had a much lower energy values than the rest of the solutions. The average degree of charge state per residue was determined using the coupling between the conformations versus the pH model. The Boltzmann average over all the conformations was completely determined by the lowest energy conformation. It was found that at pH 7 only one out of six chargeable amino acids is neutral despite the proximity of these residues among themselves (see figure 1b).

The structure of **P013 **can be briefly described as a bend in which the five negatively charged residues (**E1**, **D3**, **D4**, **D5**, **D6**) are pointing towards the solvent. The uncharged **D2 **is interacting with **M9 **which is in a cluster of residues that form a kind of hydrophobic core (**F7**, **G8**, **M9**, **G10**, **A11**, **L12 **and **F13**).

**Figure 2:**
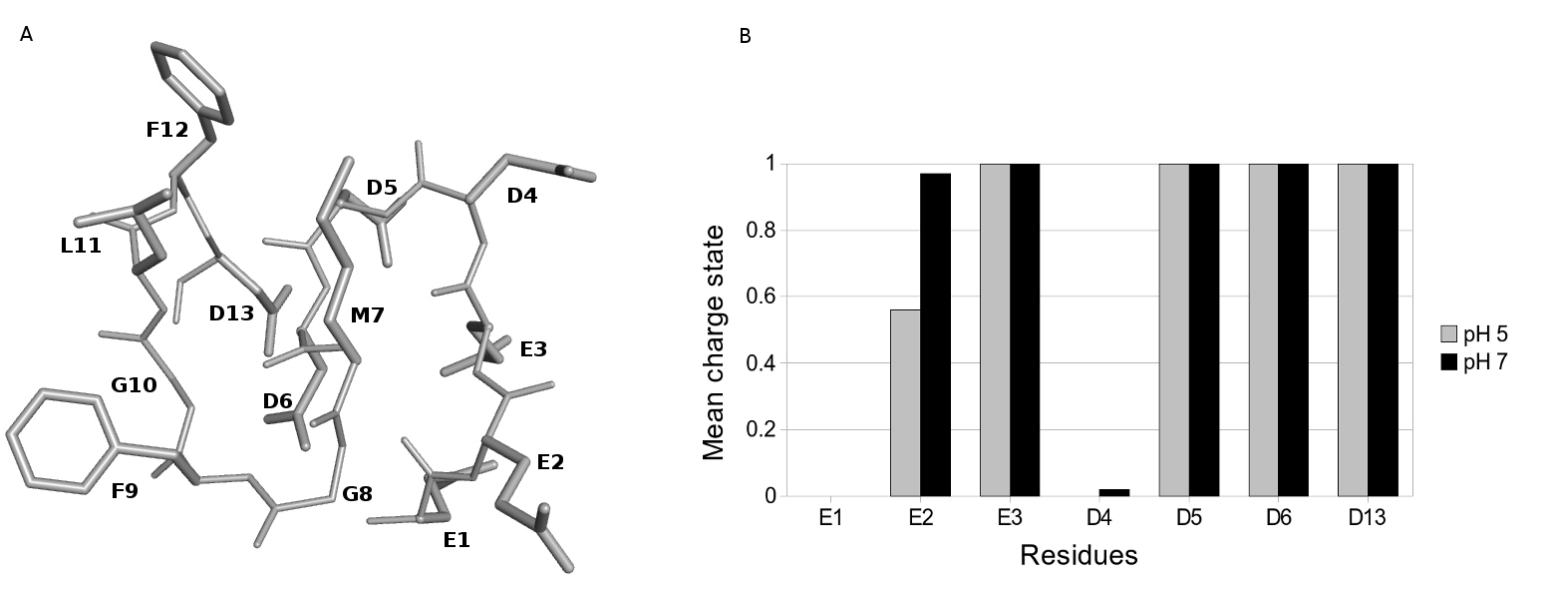
**Structure of R13 peptide**. A) Representation of the theoretical minimum energy conformation of R13 peptide. Amino acids are labelled using one letter code. B) Boltzmann average degree of charge in accord with the conformational pH model, light gray pH 5, dark grey pH 7. At both pHs values the uncharged residues are E1 and D4.

The EDMC of the **R13 **(EEEDDDMGFGLFD) can be also described as a bend. The lowest energy conformation out of the 12000 conformation sample is shown in Figure 2a. In this case, the average degree of charge state per residue at pH 5 and pH 7 was determined, showing that **E1 **and **D4 **were uncharged. The minimum energy conformations were the same at both pHs. At pH 7 the energy was slightly lower and the mean charge state was slightly different (Figure 2b). As we saw with **P013**, the result of the Boltzmann average is entirely determined by the lowest energy conformation. The reason for calculating the structure at pH 5 was to compare our results directly with the ones obtained by the NMR studies of this peptide by Soares *et al *[17]. They found that R13 behaves as a random coil in solution, and that the binding to antibody reduces the peptide flexibility, inducing a more defined conformation. We found that both, P013 and R13, are bends. It is possible to argue that the flexibility of R13 hinders the assignment of a defined structure. We could report a definite structure because in our method we only analyzed the minimum energy conformation without taking into account the flexibility of the peptides.

### 3.2. Conformational comparison of P013, R13, H13, A13 and rhodopsin

**Figure 3:**
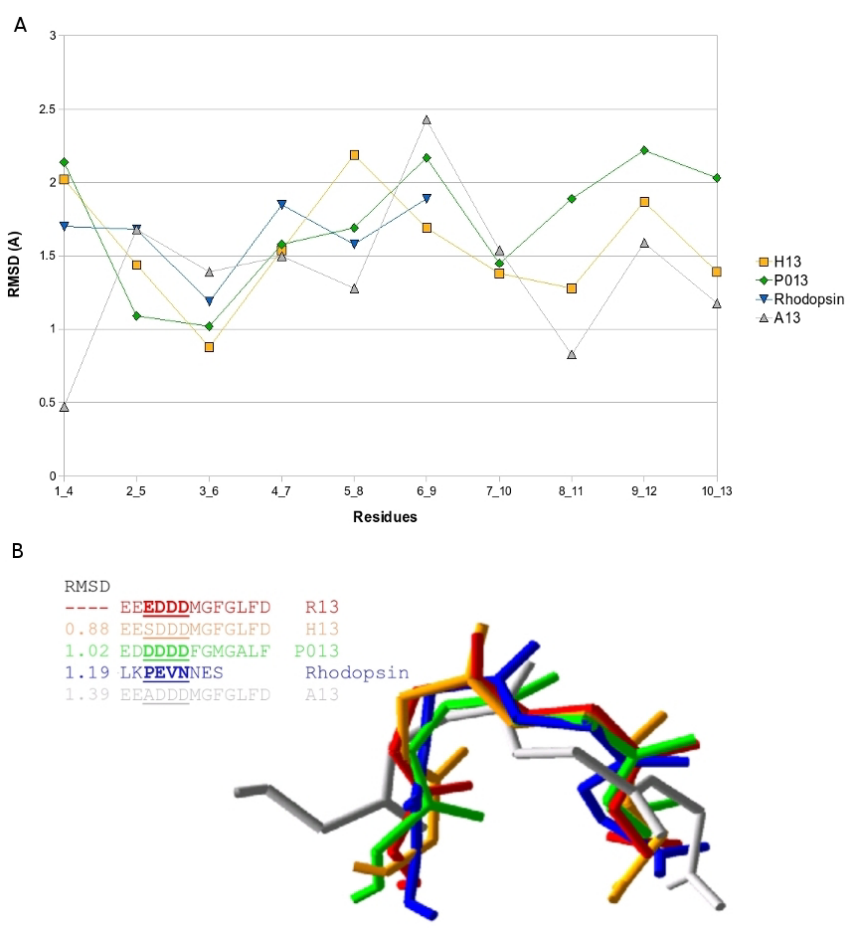
**Structural alignment of the five peptides**. A) RMSD values resulting from the structural alignment of P013 (green), H13 (orange), A13 (grey) and 2° rhodopsin loop (blue) all against R13 peptide, using a 4 residue sliding window. B) Superimposition of R13 (red), P013 (green), H13 (orange), A13 (gray) and 2° rhodopsin loop (blue). Only the underlined residues are shown (insert).

Superposition of **P013 **and **R13 **showed that they were quite similar in the curved region of the bend (residues 3 to 6) while the conformation for the rest of the residues was different. The next step was to compare them with **SEL **(the second extra-cellular loop of our homology model of human rhodopsin, based on the bovine crystal structure) and the experimental NMR structures of **H13 **(PDB code 1S4J) and **A13 **(PDB code 1S4H). The structural alignment RMSD values, against R13, can be seen in Figure 3a. The structural alignment of the five peptides showed a surprising conformational similarity to residues 3 to 6 (Figure 3b).

Experimental data demonstrated that the affinity of these peptides towards monoclonal antibody 17.2 follows the order **R13 **> **H13 **≈ **P013 **> **A13 **[14,17]. Remarkably, their conformational differences, which are reflected in the RMSD values of the structural alignment, followed an identical order suggesting that there may be a correlation between the conformation of the bend and its affinity. See figure 3.

Another important outcome was finding out that SEL13, a portion of the second extra cellular loop of rhodopsin, has a matching shape in spite of not having sequence identity with these peptides. This fact strongly reinforces the results of studies that have associated the dysfunction of the retina in chagasic patients with an autoimmune mechanism in which anti-P auto-antibodies would cross-react with the visual pigment rhodopsin [13]. Our findings also support one of the conclusions of a previous study by Soares *et al*. [17] about the importance of the conformational state in the peptide-antibody interaction.

### 3.3. Structure of antibody mAb17.2 Fv domain and Antibody-peptide docking analysis

**Figure 4:**
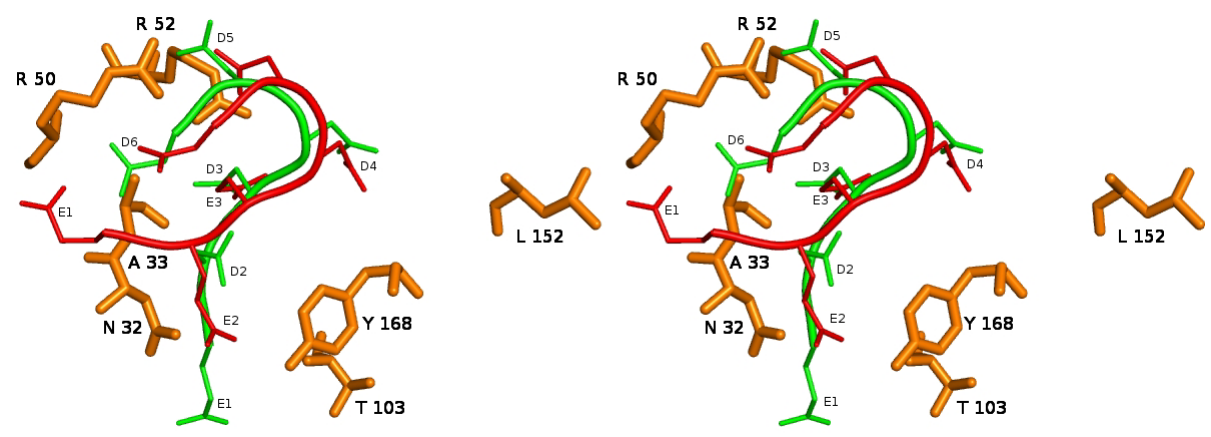
**Top-side view of R13 and P013 bonded to mAb17.2**. The antibodies residues (orange) are depicted using the stick representation and labelled using one-letter code, the most relevant residues are shown. The thin line corresponds to R13 (red) and P013 (green).

The electrostatic potential surface of the antibody revealed that the antigen-binding site, formed by the six CDR loops from the two variable chains, is a positively charged depression with a small and narrow cavity at the bottom. See Figure 4.

The docking of the R13 peptide into the antibody showed that the curve formed by **E3**, **D4**, **D5 **and **D6 **binds very deeply into the cavity while the residues forming the hydrophobic core (residues 7 to 13) were associated with the first hypervariable loop of the light chain.

**Figure 5:**
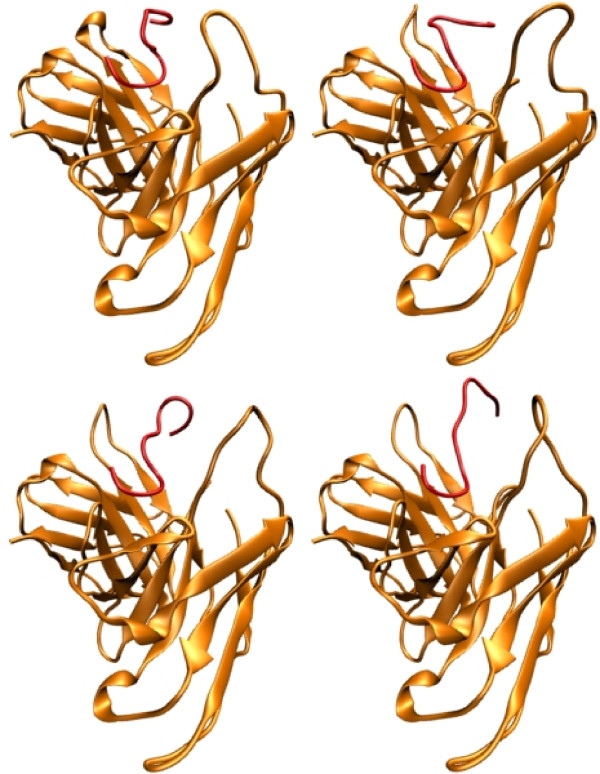
**Refinement of R13 docking results**. Cartoon representation of the result of the docking analysis and the refinement procedure. Antibody is coloured orange, and R13 peptide is red. From left to right and top to bottom, the docking result without refinement, a snapshot of the MD trajectory after one cycle of heating and and cooling, a snapshot after two cycles and finally the last snapshot after the whole procedure was done.

The most dominating feature of the docking is the neutralization of the charged residues in the peptide through the formation of ion pairs between R50 (CDR2, heavy chain) and E3 plus R52 (CDR2, heavy chain) with D5 and D13. D6 is pointing towards the solvent. See figure 4. These interactions were observed before and after the refinement process, except in the case of D13 which after the refinement, and due to the elongation of the peptide, is now pointing towards the solvent, as can be seen figure 5.

Other observed electrostatic interactions were of the hydrogen bond type. E2 backbone carbonyl is hydrogen bonded to R165 (light chain) and G101 (heavy chain). E3 hydroxyl is hydrogen bonded to Y215 hydroxyl (light chain) D4 is forming and hydrogen bond with L152 and G210 (light chain).

The docking of the P013 peptide showed that the bend residues (3 to 6) bind similarly but less deeply than R13. **D3 **and **5 **are again neutralized by **R50 **and **52 **(heavy chain). The main difference observed when comparing electrostatic interactions between these two peptides is the ion pair formed between D4, which is unprotonated in P013, and R165 (light chain). The remaining residues (7 to 13) are exposed to the solvent instead of interacting with the CDR1 of the light chain as in the case of R13.

**Figure 6:**
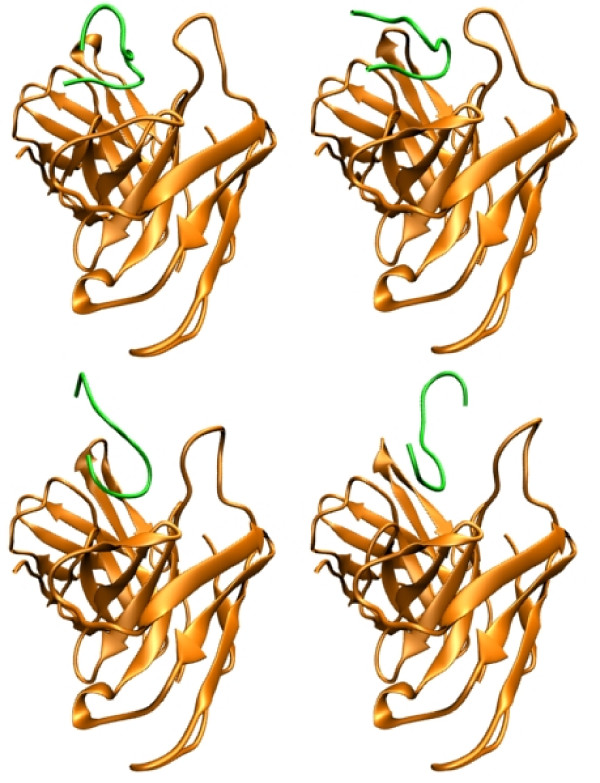
**Refinement of P013 docking results**. Cartoon representation of the result of the docking analysis and the refinement procedure. Antibody is colored orange, and P013 peptide is green. From left to right and top to bottom, the docking result without refinement, a snapshot of the MD trajectory after one cycle of heating and and cooling, a snapshot after two cycles and finally the last snapshot after the whole procedure was done.

The results obtained directly from the docking of the P013 peptide (without refinement) showed that the bend residues 3 to 6 bind similarly but less deeply than R13. After the refinement, the curved region (residues 3 to 6) becomes less curved and there is a change in the relative position of the C terminal with respect to the antibody, this is shown in figure 5. The E1 backbone carbonyl is forming a hydrogen bond with the side chain amide nitrogen of K149 (light chain). D2, D4, D5 and D6 are neutralizing K172, R165 (both at the light chain), R50 and R52 (both at the heavy chain), respectively. The remaining residues (7 to 13) are exposed to the solvent. See figure 6.

Another difference observed between the interactions of the two peptides with the antibody is the variation of the solvent accessible surface between the free and the docked state. The change in the solvent accessible surface was 10% for P013 and 13% for R13 after refinement. This parameter is a measure of the entropy increase in the complex formation and is given by the water molecule exclusion in the interaction surface.

The third docking study was about rhodopsin (this complex was not refined due to the size of the system). It was found that the residues **PEVN **of the second extra cellular loop bind in a similar fashion as to residues 3 to 6 in the R13-P013 complexes. Rhodopsin **E197 **is occupying the same position as **E3 **in the R13 complex. It is stabilized by the formation of two ions pairs: the first with **R50 **(heavy chain), remaining in the same conformation as it had in the previous complexes, and the second with **R52 **(heavy chain), which has moved from the position it had in the R13 complex being now in a conformation that allows the interaction with **E197**.

## 4. Discussion

The first part of the study concerning the calculation of peptide structures by theoretical methods and their comparison with existing NMR and crystal structures reveals a most important common feature. Namely the curve formed by residues 3 to 6 having a surprising conformational similarity. Remarkably, the affinity of these peptides towards the antibody, following the order **R13 **> **H13 **≈ **P013 **> **A13 **according to previous experimental work of Smulski et al [14] and Soares et al [17], is reflected in the order of the conformational differences of the peptides with respect to R13, according to the present work.

**Figure 7:**
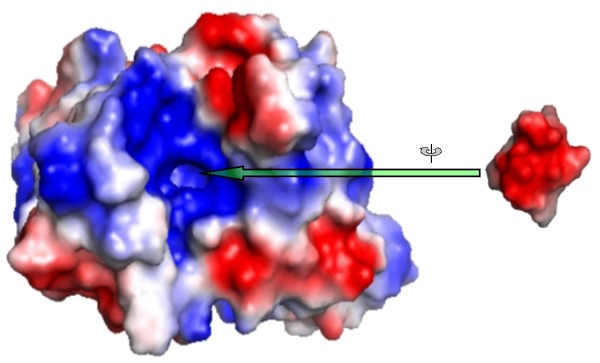
**Top view of mAb 17.2 Fv model**. The van der Waals surface is coloured according to the electrostatic potential calculated with the program Poisson-Boltzman electrostatics calculated using APBS [46] as implemented in PyMol, using default charge values and dielectric constant 80 (Receptor coloured by calculated charge from red -1 to blue +1). The binding site of the peptide is a positive charged cavity. Inferior view of R13. The peptide has been moved off the binding site and rotated 180 degrees.

In the second part of our work, which involved the modelling of the mAb17.2 Fv domain, based on the crystal structure of a high sequence identity auto-antibody to single stranded DNA, the most remarkable feature was the positively charged, narrow and deep cavity in the active site fitting the bend formed by residues 3 to 6 [46]. See figure 7.

**Figure 8:**
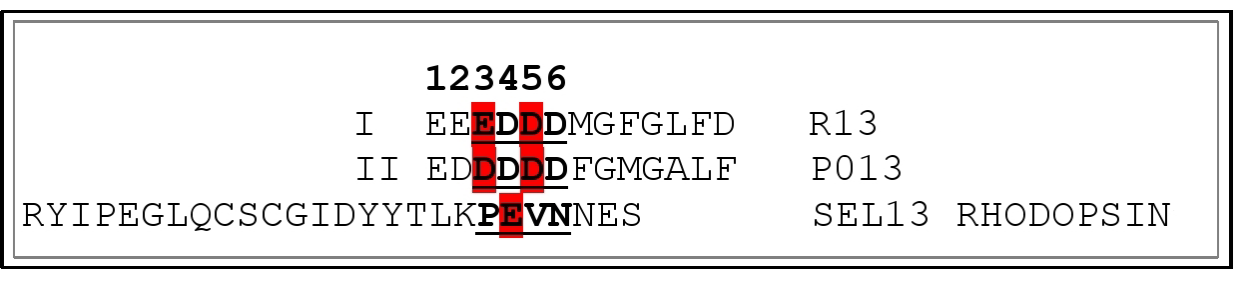
**Relevant binding residues**. Relevant binding residues found for the binding of P013, R13 peptide and Rhodopsin to mAb 17.2 are highlighted in red.

The third and final part of this study was the docking of R13, P013 and rhodopsin into the mAb17.2 Fv homology model. The analysis of the interactions indicates the residues which may play a crucial part in the process of the molecular recognition. The results suggest that residues 3 to 5 might act as an anchor. Once the bend is bound, the rest of the peptide may undergo major conformational changes allowing a tighter binding in an induced-fit fashion. P013 (before and after the refinement) seems to bind more loosely than R13. This fact is in qualitative accordance with the experimental measured affinities. Finally, the docking of rhodopsin shows that the only charged residue in the loop, **E197**, is occupying the place of **E3 **and is stabilized by **R50 **and **52**. Our model suggests that the most relevant elements in the molecular recognition of the peptides by the antibody are the shape or conformation of the bend combined with the presence of negatively charged residues in position 3 and 5 (P013 and R13), or one in position 4, as it is the case in rhodopsin, figure 8. Our results support the existence of the anti-P auto-antibodies cross-reaction and provide a "molecular *in silico *characterization" for previously reported experiments.

## 5. Conclusion

The outcome from this study clearly describes the interactions of the structural elements involved in the autoimmune mechanism of anti-P auto-antibodies cross-reaction, the stimulation of the β1-adrenoreceptor and the visual pigment rhodopsin. It also gives us some qualitative ideas to judge the potential binding of the anti-P auto-antibodies to receptor loops in different tissues. Results from this study may eventually lead to the development of treatments to abolish receptor mediated symptoms of Chagas's disease and other autoimmune pathologies.
